# Crystal structure and Hirshfeld surface analysis of 4-{2,2-di­chloro-1-[(*E*)-(4-chloro­phen­yl)diazen­yl]ethen­yl}-*N*,*N*-di­methyl­aniline

**DOI:** 10.1107/S2056989020007549

**Published:** 2020-06-09

**Authors:** Zeliha Atioğlu, Mehmet Akkurt, Namiq Q. Shikhaliyev, Sevinc H. Mukhtarova, Gulnar T. Suleymanova, Flavien A. A. Toze

**Affiliations:** a İlke Education and Health Foundation, Cappadocia University, Cappadocia Vocational College, The Medical Imaging Techniques Program, 50420 Mustafapaşa, Ürgüp, Nevşehir, Turkey; b Department of Physics, Faculty of Sciences, Erciyes University, 38039 Kayseri, Turkey; c Organic Chemistry Department, Baku State University, Z. Khalilov str. 23, AZ 1148 Baku, Azerbaijan; d Department of Chemistry, Faculty of Sciences, University of Douala, PO Box 24157, Douala, Republic of , Cameroon

**Keywords:** crystal structure, non-covalent inter­actions, azo dye, Hirshfeld surface analysis

## Abstract

The title compound, C_16_H_14_Cl_3_N_3_, comprises three mol­ecules of similar shape in the asymmetric unit. The crystal cohesion is ensured by inter­molecular C—H⋯N and C—H⋯Cl hydrogen bonds in addition to C—Cl⋯π inter­actions. Hirshfeld surface analysis and two-dimensional fingerprint plots reveal that Cl⋯H/H⋯Cl (33.6%), H⋯H (27.9%) and C⋯H/H⋯C (17.6%) are the most important contributors towards the crystal packing.

## Abstract 2

The asymmetric unit of the title compound comprises three independent mol­ecules of similar geometry. The crystal structure is stabilized by inter­molecular C—H⋯N and C—H⋯Cl hydrogen bonds in addition to C—Cl⋯π inter­actions.

## Chemical context   

Non-covalent inter­actions, such as hydrogen bonds, halogen–halogen or chalcogen–chalcogen bonds, van der Waals inter­actions or π–π stacking, π⋯cation and π⋯anion inter­actions, *etc*. are much weaker than covalent bonds. Nevertheless, they can control the reactivity of mol­ecules, the crystal packing, tautomerization and other properties (Asadov *et al.*, 2016 ▸; Mahmudov *et al.*, 2019 ▸). For example, such kinds of weak inter­actions can create inter­esting supra­molecular networks in coordination compounds, involving monomeric, oligomeric or polymeric subunits, which affects their catalytic activity (Afkhami *et al.*, 2017 ▸; Gurbanov *et al.*, 2018 ▸).
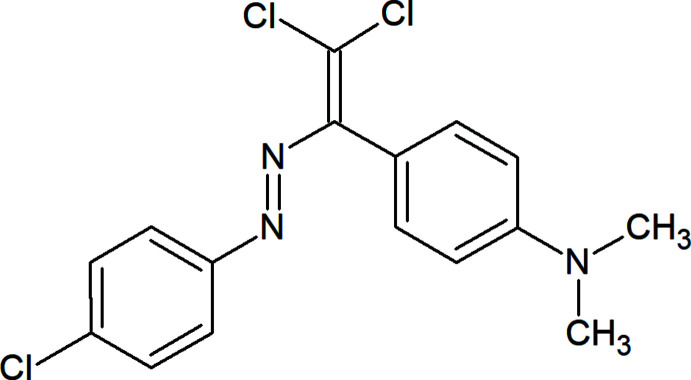


In a previous study we have attached resonance-assisted hydrogen-bonded synthons or chlorine atoms to dye mol­ecules, which leads to inter­molecular weak inter­actions for the resulting products with inter­esting analytical and solvatochromic properties (Maharramov *et al.*, 2018 ▸; Mahmudov & Pombeiro, 2016 ▸). In a continuation of our work in this direction, we now have synthesized a new azo dye, 4-{2,2-di­chloro-1-[(*E*)-(4-chloro­phen­yl)diazen­yl]ethen­yl}-*N*,*N*-di­methyl­aniline, which features C—H⋯N, C—H⋯π and C—Cl⋯Cl types of weak inter­molecular inter­actions.

## Structural commentary   

**Figure 1:**
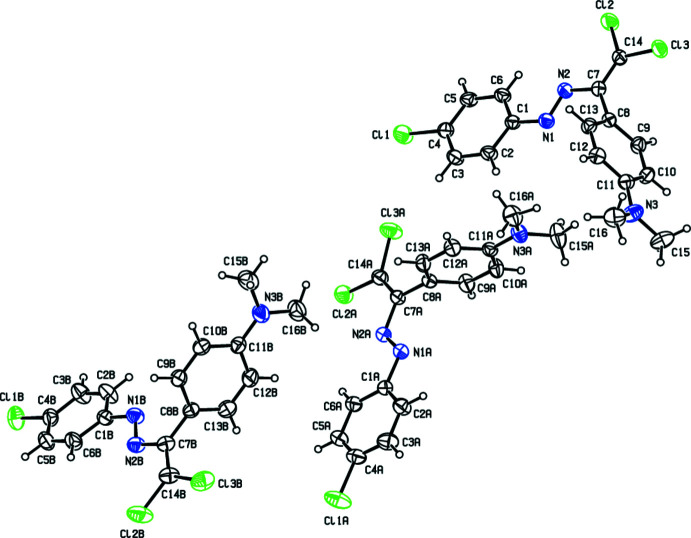
The mol­ecular structures of the three mol­ecules in the asymmetric unit of the title compound, showing the atom labelling and displacement ellipsoids drawn at the 30% probability level.

**Figure 2:**
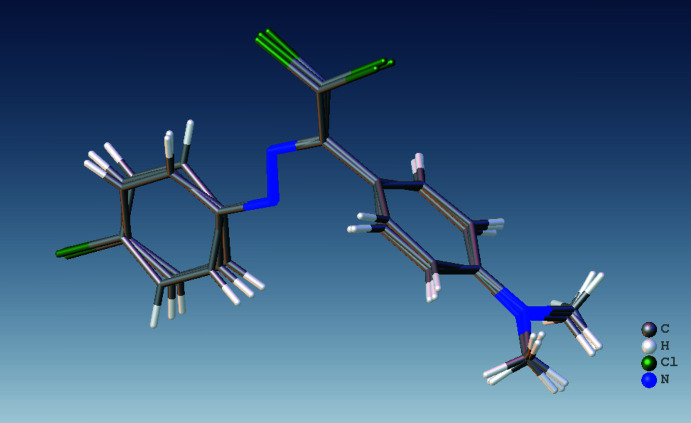
Overlay image of the three mol­ecules in the asymmetric unit of the title compound.

The asymmetric unit of the title compound (Fig. 1 ▸) contains three mol­ecules of similar shape, hereafter referred to as Mol-N1 (C1–C16/N1–N3/Cl1–Cl3), Mol-N1*A* (C1*A*–C16*A*/N1*A*–N3*A*/Cl1*A*–Cl3*A*) and Mol-N1*B* (C1*B*–C16*B*/N1*B*–N3*B*/Cl1*B*–Cl3*B*). The conformational differences between mol­ecules Mol-N1, Mol-N1*A* and Mol-N1*B* are highlighted in an overlay diagram shown in Fig. 2 ▸. The dihedral angles between the benzene rings [C1–C6 and C8–C13 (mol­ecule Mol-N1), C1*A*–C6*A* and C8*A*–C13*A* (mol­ecule Mol-N1*A*), and C1*B*–C6*B* and C8*B*–C13*B* (mol­ecule Mol-N1*B*)] of the 4-chloro­phenyl and *N*,*N*-di­methyl­aniline groups are 69.94 (10), 79.68 (12) and 88.08 (13)°, respectively. In mol­ecule Mol-N1, the N1—N2—C7—C14, N2—C7—C14—Cl2, N2—C7—C14—Cl3 and C8—C7—C14—Cl3 torsion angles are −178.7 (2), 3.1 (3), −176.21 (16) and 4.1 (3)°, respectively. The corres­ponding angles are 178.4 (2), 3.8 (3), −175.1 (2) and 2.5 (3)° for mol­ecule Mol-N1*A*, and −175.0 (2), 0.3 (3), 179.71 (18) and −0.1 (4) for mol­ecule Mol-N1*B*.

## Supra­molecular features and Hirshfeld surface analysis   

**Figure 3:**
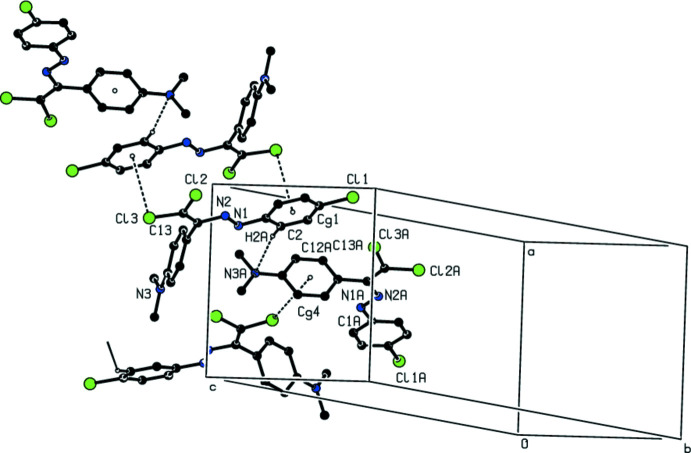
A partial view of the crystal packing of the title compound. Inter­molecular inter­actions are shown as dashed lines.

In the crystal, the mol­ecules are connected by inter­molecular C—H⋯N and C—H⋯Cl hydrogen bonds and C—Cl⋯π inter­actions, which contribute to the overall packing, forming a three-dimensional network (Table 1 ▸; Fig. 3 ▸).

**Figure 4:**
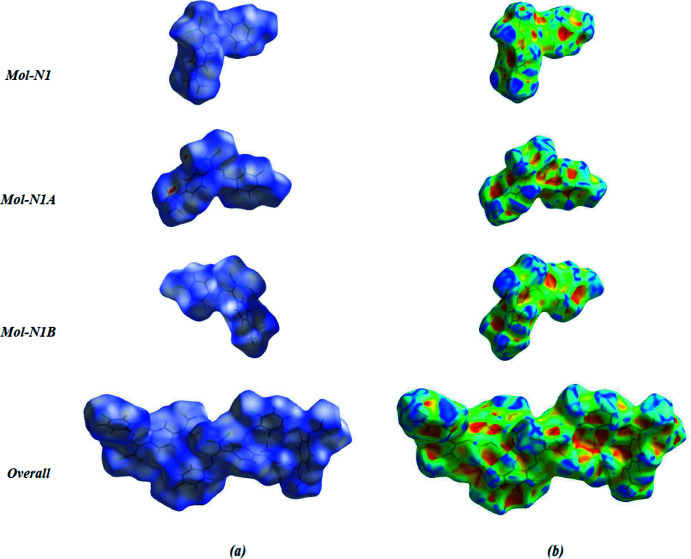
Hirshfeld surface of the title compound (symmetry-independent mol­ecules Mol-N1, Mol-N1*A* and Mol-N1*B*, and overall), with (*a*) the inter­action of neighbouring mol­ecules mapped over *d*_norm_ and (*b*) mapped over shape-index.

Hirshfeld surface analysis was used to investigate the presence of hydrogen bonds and inter­molecular inter­actions in the crystal structure. The Hirshfeld surfaces (Spackman & Jayatilaka, 2009 ▸) and the associated two-dimensional fingerprint plots (McKinnon *et al.*, 2007 ▸) of the title compound were calculated using *Crystal Explorer 17.5* (Turner *et al.*, 2017 ▸). The three-dimensional mol­ecular Hirshfeld surfaces of the three mol­ecules Mol-N1, Mol-N1*A* and Mol-N1*B* and the overall surface were generated using a high standard surface resolution colour-mapped over the normalized contact distance. The red, white and blue regions visible on the *d*_norm_ surfaces indicate contacts with distances shorter, longer and equal to the van der Waals radii (Fig. 4 ▸ *a*). The shape-index of the Hirshfeld surface is a tool to visualize π–π stacking inter­actions; Fig. 4 ▸ *b* clearly suggest that there are no π–π inter­actions in the title compound. The red spots in Fig. 4 ▸ *a* correspond to the relatively strong C—H⋯N hydrogen-bonding inter­actions in the crystal structure; in Mol-N1*A* it involves the N3*A* atoms of the *N*,*N*-di­methyl­aniline group as acceptors with the aromatic H2*A* donor atom of the chloro­benzene ring in Mol-N1 (C2—H2*A*⋯N3*A*).

**Figure 5:**
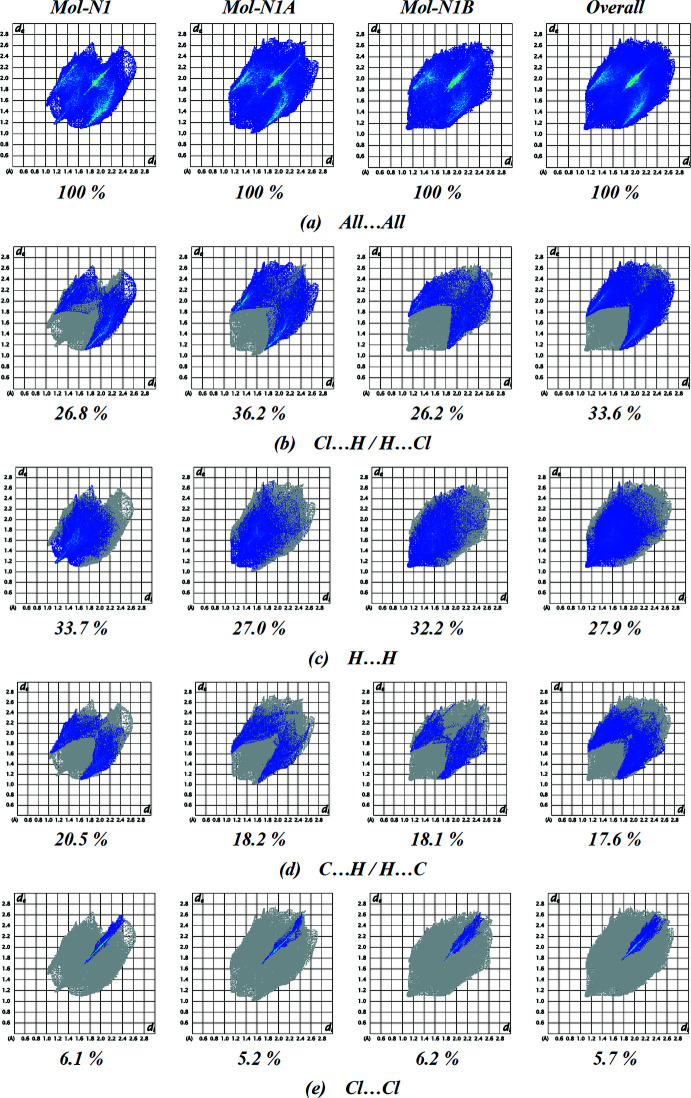
Fingerprint plots representative of specific inter­atomic contacts in the title compound (symmetry-independent mol­ecules Mol-N1, Mol-N1*A*, Mol-N1*B* and overall), (*a*) for all inter­actions, and delineated into (*b*) Cl⋯H/H⋯Cl, (*c*) H⋯H, (*d*) C⋯H/H⋯C and (*e*) Cl⋯Cl inter­actions.

Two-dimensional fingerprint plots are presented in Fig. 5 ▸. The red points, which represent closer contacts and negative *d*_norm_ values on the surface, correspond to C—H⋯Cl inter­actions. The reciprocal Cl⋯H/H⋯Cl inter­actions appear as two symmetrical broad wings with *d*_e_ + *d*_i_ ≃ 2.85 Å and contribute 33.6% to the Hirshfeld surface (Fig. 5 ▸ *b*). Another significant reciprocal inter­action (H⋯H) with a contribution of 27.9% is present as broad symmetrical spikes at diagonal axes *d*_e_ + *d*_i_ ≃ 2.2 Å (Fig. 5 ▸ *c*). The pair of characteristic wings in the fingerprint plot delineated into C⋯H/H⋯C contacts (Tables 2 ▸ and 3 ▸, Fig. 5 ▸ *d*; 17.6% contribution to the Hirshfeld surface), have tips at *d*_e_ + *d*_i_ ≃ 2.80 Å. The Cl⋯Cl contacts, Fig. 5 ▸ *e* (5.7% contribution), have an arrow-shaped distribution of points with the tip at *d*_e_ = *d*_i_ = 3.50 Å.

The other weak inter­molecular inter­actions, *viz*. Cl⋯C/C⋯Cl (5.4%), N⋯H/H⋯N (4.7%), C⋯C (1.7%), Cl⋯N/N⋯Cl (1.6%), N⋯C/C⋯N (1.0%) and N⋯N (0.8%) contacts, show only small contributions and thus have a negligible effect on the packing.

## Database survey   

A search of the Cambridge Structural Database (CSD, Version 5.40, November 2018; Groom *et al.*, 2016 ▸) for structures having an (*E*)-1-(2,2-di­chloro-1-phenyl­vin­yl)-2-phenyl­diazene skeleton gave 25 hits, of which six closely resemble the title compound, *viz*. 1-(4-bromo­phen­yl)-2-[2,2-di­chloro-1-(4-nitro­phen­yl)ethen­yl]diazene (CSD refcode HONBOE; Akkurt *et al.*, 2019 ▸), 1-(4-chloro­phen­yl)-2-[2,2-di­chloro-1-(4-nitro­phen­yl)ethen­yl]diazene (HONBUK; Akkurt *et al.*, 2019 ▸), 1-(4-chloro­phen­yl)-2-[2,2-di­chloro-1-(4-fluoro­phen­yl)ethen­yl]diazene (HODQAV; Shixaliyev *et al.*, 2019 ▸), 1-[2,2-di­chloro-1-(4-nitro­phen­yl)ethen­yl]-2-(4-fluoro­phen­yl)diazene (XIZ­REG; Atioğlu *et al.*, 2019 ▸), 1,1-[methyl­enebis(4,1-phenyl­ene)]bis­[(2,2-di­chloro-1-(4-nitro­phen­yl)ethen­yl]diazene (LEQ­XIR; Shixaliyev *et al.*, 2018 ▸), 1,1-[methyl­enebis(4,1-phenyl­ene)]bis­{[2,2-di­chloro-1-(4-chloro­phen­yl) ethen­yl]diazene} (LEQXOX; Shixaliyev *et al.*, 2018 ▸).

In the crystal structures of HONBOE and HONBUK, the aromatic rings form dihedral angles of 60.9 (2) and 64.1 (2)°, respectively. Mol­ecules are linked through weak *X*⋯Cl contacts [*X* = Br for HONBOE, and Cl for HONBUK], C—H⋯Cl and C—Cl⋯π inter­actions into sheets parallel to (001). Additional van der Waals inter­actions consolidate the three-dimensional packing. In the crystal of HODQAV, mol­ecules are stacked in columns along [100] *via* weak C—H⋯Cl hydrogen bonds and face-to-face π–π stacking inter­actions. The crystal packing is further stabilized by short Cl⋯Cl contacts. In XIZREG, mol­ecules are linked by C—H⋯O hydrogen bonds into zigzag chains running along [001]. The crystal packing is further stabilized by C—Cl⋯π, C—F⋯π and N—O⋯π inter­actions. In the crystal of LEQXIR, C—H⋯N and C—H⋯O hydrogen bonds and Cl⋯O contacts were found, and in LEQXOX, C—H⋯N and Cl⋯Cl contacts are observed.

## Synthesis and crystallization   

The title compound was synthesized according to a reported literature protocol (Maharramov *et al.*, 2018 ▸). A 20 ml screw-neck vial was charged with DMSO (10 ml), (*E*)-4-[(2-(4-chloro­phen­yl)hydrazineyl­idene]meth­yl)-*N*,*N*-di­methyl­aniline (274 mg, 1 mmol), tetra­methyl­ethylenedi­amine (TMEDA) (295 mg, 2.5 mmol), CuCl (2 mg, 0.02 mmol) and CCl_4_ (20 mmol, 10 equiv). After 1–3 h (until TLC analysis showed complete consumption of the corresponding Schiff base), the reaction mixture was poured into a ∼0.01 *M* solution of HCl (100 mL, pH = ∼2–3) and extracted with di­chloro­methane (3 × 20 ml). The combined organic phase was washed with water (3 x 50 ml), brine (30 ml), dried over anhydrous Na_2_SO_4_ and concentrated *in vacuo* in a rotary evaporator. The residue was purified by column chromatography on silica gel using appropriate mixtures of hexane and di­chloro­methane (3/1–1/1) to give an orange solid. Yield: 72%; mp 408 K. Analysis: calculated for C_16_H_14_Cl_3_N_3_: C 54.19, H 3.98, N 11.85; found: C 54.08, H 3.91, N 11.82%. ^1^H NMR (300 MHz, CDCl_3_) δ 3.05 (6H, NMe_2_), 6.79–7.79 (8H, Ar). ^13^C NMR (75 MHz, CDCl_3_) δ 152.41, 151.45, 150.29, 137.26, 135.11, 131.08, 129.27, 124.50, 119.11, 111.48, 40.29. ESI–MS: *m*/*z*: 355.48 [*M* + H]^+^.

Crystals suitable for X-ray analysis were obtained by slow evaporation of an ethanol solution.

## Refinement   

Crystal data, data collection and structure refinement details are summarized in Table 3 ▸. All C-bound H atoms were refined using a riding model with *d*(C—H) = 0.93 Å, *U_i_*_so_(H) = 1.2*U*_eq_(C) for aromatic H atoms, and 0.96 Å, *U*_iso_(H) = 1.5*U*_eq_(C) for methyl H atoms. Owing to poor agreement between observed and calculated intensities, five outliers (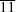 0 4), (4 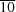 13), (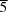 8 8), (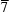 2 18) and (1 8 14) were omitted in the final cycles of refinement.

## Supplementary Material

Crystal structure: contains datablock(s) I. DOI: 10.1107/S2056989020007549/wm5554sup1.cif

Structure factors: contains datablock(s) I. DOI: 10.1107/S2056989020007549/wm5554Isup2.hkl

Supporting information file. DOI: 10.1107/S2056989020007549/wm5554Isup3.cml

CCDC reference: 2007970

Additional supporting information:  crystallographic information; 3D view; checkCIF report

## Figures and Tables

**Table 1:** Hydrogen-bond geometry (Å, °) *Cg*1 and *Cg*4 are the centroids of the C1–C6 and C8*A*–C13*A* rings, respectively.

*D*—H⋯*A*	*D*—H	H⋯*A*	*D*⋯*A*	*D*—H⋯*A*
C2—H2*A*⋯N3*A*	0.93	2.68	3.597 (3)	167
C5*B*—H5*BA*⋯Cl3^i^	0.93	2.95	3.703 (3)	139
C14—Cl3⋯*Cg*1^ii^	1.71 (1)	3.55 (1)	4.083 (2)	96 (1)
C14*B*—Cl3*B*⋯*Cg*4^iii^	1.71 (1)	3.85 (1)	5.300 (3)	142 (1)

Symmetry codes: (i) 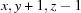; (ii) 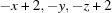; (iii) 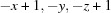.

**Table 2:** Summary of short inter­atomic contacts (Å) in the title compound

Contact	Distance	Symmetry operation
(C2) H2*A*⋯N3*A* (C11*A*)	2.68	(*x*, *y*, *z*)
(C4) Cl1⋯Cl1*B* (C4*B*)	3.5403 (11)	(2 − *x*, 2 − *y*, 1 − *z*)
(C14) Cl2⋯Cl1 (C4)	3.6580 (11)	(2 − *x*, 1 − *y*, 2 − *z*)
(C13) H13*A*⋯Cl2 (C14)	3.10	(2 − *x*, −*y*, 2 − *z*)
(C14) Cl3⋯H5*BA* (C5*B*)	2.95	(*x*, −1 + *y*, 1 + *z*)
(C9) H9*A*⋯H15*D* (C15*A*)	2.60	(1 − *x*, −*y*, 2 − *z*)
(C15) H15*C*⋯Cl3 (C14)	3.00	(1 − *x*, −1 − *y*, 2 − *z*)
(C4) C5⋯H12*A* (C12)	2.95	(*x*, 1 + *y*, *z*)
(C6*A*) H6*AA*⋯H12*C* (C12*B*)	2.54	(*x*, *y*, *z*)
(C5*A*) H5*AA*⋯Cl2*A* (C14*A*)	3.10	(1 − *x*, 1 − *y*, 1 − *z*)
(C9*A*) H9*AA*⋯N2*B* (N1*B*)	2.92	(1 − *x*, 1 − *y*, 1 − *z*)
(C11*A*) N3*A*⋯H2*A* (C2)	2.68	(*x*, *y*, *z*)
(C14*A*) Cl3*A*⋯H16*E* (C16*A*)	3.09	(*x*, 1 + *y*, *z*)
(C14*A*) Cl3*A*⋯Cl1*B* (C4*B*)	3.6816 (11)	(2 − *x*, 2 − *y*, 1 − *z*)
(C4*A*) C5*A*⋯H15*G* (C15*B*)	2.97	(−1 + *x*, *y*, *z*)
(C3*A*) H3*AA*⋯H16*I* (C16*B*)	2.49	(1 − *x*, −*y*, 1 − *z*)
(C15*A*) H15*D*⋯H9*A* (C9)	2.60	(1 − *x*, −*y*, 2 − *z*)
(C12*A*) H12*B*⋯C4*B* (Cl1*B*)	2.98	(2 − *x*, 1 − *y*, 1 − *z*)
(C4*B*) Cl1*B*⋯Cl1 (C4)	3.5403 (11)	(2 − *x*, 2 − *y*, 1 − *z*)
(C4*B*) Cl1*B*⋯Cl3*A* (C14*A*)	3.6816 (11)	(2 − *x*, 2 − *y*, 1 − *z*)
(Cl1*B*) C4*B*⋯H12*B* (C12*A*)	2.98	(2 − *x*, 1 − *y*, 1 − *z*)
(C16*B*) H16*I*⋯H3*AA* (C3*A*)	2.49	(1 − *x*, −*y*, 1 − *z*)
(N1*B*) N2*B*⋯H9*AA* (C9*A*)	2.92	(1 − *x*, 1 − *y*, 1 − *z*)
(C8*B*) C9*B*⋯H3*BA* (C3*B*)	2.92	(*x*, −1 + *y*, *z*)
(C15*B*) H15*G*⋯C5*A* (C4*A*)	2.97	(1 + *x*, *y*, *z*)
(C15*B*) H15*I*⋯H2*BA* (C2*B*)	2.37	(2 − *x*, 1 − *y*, 1 − *z*)
(C12*B*) H12*C*⋯H6*AA* (C6*A*)	2.54	(*x*, *y*, *z*)
(C5*B*) H5*BA*⋯Cl3 (C14)	2.95	(*x*, 1 + *y*, −1 + *z*)

**Table 3:** Experimental details

Crystal data	
Chemical formula	C_16_H_14_Cl_3_N_3_
*M*_r_	354.65
Crystal system, space group	Triclinic, *P* 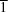
Temperature (K)	296
*a*, *b*, *c* (Å)	9.7515 (5), 9.8203 (5), 26.6696 (16)
α, β, γ (°)	92.338 (2), 91.212 (2), 94.048 (2)
*V* (Å^3^)	2544.7 (2)
*Z*	6
Radiation type	Mo *K*α
μ (mm^−1^)	0.54
Crystal size (mm)	0.24 × 0.15 × 0.09

Data collection	
Diffractometer	Bruker APEXII PHOTON 100 detector
Absorption correction	Multi-scan (*SADABS*; Krause *et al.*, 2015 ▸)
*T*_min_, *T*_max_	0.894, 0.946
No. of measured, independent and observed [*I* > 2σ(*I*)] reflections	40829, 9634, 6689
*R*_int_	0.056
(sin θ/λ)_max_ (Å^−1^)	0.610

Refinement	
*R*[*F* ^2^ > 2σ(*F* ^2^)], *wR*(*F* ^2^), *S*	0.041, 0.115, 1.01
No. of reflections	9634
No. of parameters	601
H-atom treatment	H-atom parameters constrained
Δρ_max_, Δρ_min_ (e Å^−3^)	0.29, −0.30

Computer programs: *APEX3* and *SAINT* (Bruker, 2007 ▸), *SHELXT* (Sheldrick, 2015*a* ▸), *SHELXL* (Sheldrick, 2015*b* ▸), *ORTEP-3 for Windows* (Farrugia, 2012 ▸), *OLEX2* (Dolomanov *et al.*, 2009 ▸), *PLATON* (Spek, 2020 ▸) and *publCIF* (Westrip, 2010 ▸).

## supplementary crystallographic information

### Crystal data

**Table:**

C_16_H_14_Cl_3_N_3_	*Z* = 6
*M**_r_* = 354.65	*F*(000) = 1092
Triclinic, *P*1	*D*_x_ = 1.389 Mg m^−^^3^
*a* = 9.7515 (5) Å	Mo *K*α radiation, λ = 0.71073 Å
*b* = 9.8203 (5) Å	Cell parameters from 9939 reflections
*c* = 26.6696 (16) Å	θ = 2.3–25.6°
α = 92.338 (2)°	µ = 0.54 mm^−^^1^
β = 91.212 (2)°	*T* = 296 K
γ = 94.048 (2)°	Plate, orange
*V* = 2544.7 (2) Å^3^	0.24 × 0.15 × 0.09 mm

### Data collection

**Table:**

Bruker APEXII PHOTON 100 detector diffractometer	6689 reflections with *I* > 2σ(*I*)
φ and ω scans	*R*_int_ = 0.056
Absorption correction: multi-scan (*SADABS*; Krause *et al.*, 2015)	θ_max_ = 25.7°, θ_min_ = 2.3°
*T*_min_ = 0.894, *T*_max_ = 0.946	*h* = −11→11
40829 measured reflections	*k* = −11→11
9634 independent reflections	*l* = −32→32

### Refinement

**Table:**

Refinement on *F*^2^	0 restraints
Least-squares matrix: full	Hydrogen site location: inferred from neighbouring sites
*R*[*F*^2^ > 2σ(*F*^2^)] = 0.041	H-atom parameters constrained
*wR*(*F*^2^) = 0.115	*w* = 1/[σ^2^(*F*_o_^2^) + (0.050*P*)^2^ + 0.8216*P*] where *P* = (*F*_o_^2^ + 2*F*_c_^2^)/3
*S* = 1.01	(Δ/σ)_max_ = 0.001
9634 reflections	Δρ_max_ = 0.29 e Å^−^^3^
601 parameters	Δρ_min_ = −0.30 e Å^−^^3^

### Special details

**Table:**

Geometry. All esds (except the esd in the dihedral angle between two l.s. planes) are estimated using the full covariance matrix. The cell esds are taken into account individually in the estimation of esds in distances, angles and torsion angles; correlations between esds in cell parameters are only used when they are defined by crystal symmetry. An approximate (isotropic) treatment of cell esds is used for estimating esds involving l.s. planes.

### Fractional atomic coordinates and isotropic or equivalent isotropic displacement parameters (Å^2^)

**Table:**

	*x*	*y*	*z*	*U*_iso_*/*U*_eq_	
C1	0.8482 (2)	0.1999 (2)	0.93028 (8)	0.0452 (5)	
C2	0.8218 (2)	0.1942 (2)	0.87896 (8)	0.0546 (6)	
H2A	0.774145	0.117448	0.863853	0.066*	
C3	0.8656 (3)	0.3016 (2)	0.85007 (9)	0.0572 (6)	
H3A	0.848414	0.297623	0.815595	0.069*	
C4	0.9351 (2)	0.4144 (2)	0.87313 (9)	0.0524 (5)	
C5	0.9607 (2)	0.4232 (2)	0.92419 (9)	0.0539 (6)	
H5A	1.006727	0.501099	0.939152	0.065*	
C6	0.9173 (2)	0.3156 (2)	0.95289 (8)	0.0511 (5)	
H6A	0.934411	0.320453	0.987379	0.061*	
C7	0.7902 (2)	−0.0305 (2)	1.02733 (8)	0.0475 (5)	
C8	0.7076 (2)	−0.1457 (2)	1.00137 (8)	0.0445 (5)	
C9	0.5679 (2)	−0.1675 (2)	1.00872 (9)	0.0534 (6)	
H9A	0.526051	−0.111602	1.031902	0.064*	
C10	0.4892 (2)	−0.2699 (2)	0.98260 (9)	0.0557 (6)	
H10A	0.395461	−0.281093	0.988356	0.067*	
C11	0.5471 (2)	−0.3571 (2)	0.94773 (8)	0.0498 (5)	
C12	0.6895 (2)	−0.3368 (2)	0.94155 (9)	0.0519 (5)	
H12A	0.732733	−0.394639	0.919443	0.062*	
C13	0.7660 (2)	−0.2337 (2)	0.96741 (8)	0.0507 (5)	
H13A	0.859983	−0.222405	0.962023	0.061*	
C14	0.8254 (2)	−0.0283 (2)	1.07589 (8)	0.0509 (5)	
C15	0.3225 (3)	−0.4714 (3)	0.92575 (14)	0.0888 (9)	
H15A	0.283755	−0.384431	0.924067	0.133*	
H15B	0.304255	−0.508699	0.957852	0.133*	
H15C	0.281807	−0.532564	0.899690	0.133*	
C16	0.5316 (3)	−0.5541 (3)	0.88809 (12)	0.0829 (8)	
H16A	0.588830	−0.508824	0.864106	0.124*	
H16B	0.461159	−0.612219	0.870729	0.124*	
H16C	0.586559	−0.608226	0.908655	0.124*	
N1	0.79963 (19)	0.08299 (18)	0.95616 (7)	0.0504 (4)	
N2	0.83684 (19)	0.08700 (19)	1.00163 (7)	0.0508 (4)	
N3	0.4692 (2)	−0.4541 (2)	0.91918 (9)	0.0725 (6)	
Cl1	0.99248 (9)	0.55056 (8)	0.83780 (3)	0.0881 (3)	
Cl2	0.91333 (8)	0.10675 (7)	1.10783 (2)	0.0745 (2)	
Cl3	0.78721 (7)	−0.16465 (7)	1.11246 (2)	0.06280 (17)	
C1A	0.4229 (2)	0.1795 (2)	0.57132 (8)	0.0498 (5)	
C2A	0.3627 (3)	0.0499 (3)	0.56121 (9)	0.0640 (6)	
H2AA	0.357155	−0.012520	0.586525	0.077*	
C3A	0.3104 (3)	0.0118 (3)	0.51352 (11)	0.0718 (7)	
H3AA	0.268621	−0.075308	0.506822	0.086*	
C4A	0.3211 (3)	0.1042 (3)	0.47640 (9)	0.0646 (7)	
C5A	0.3798 (3)	0.2340 (3)	0.48587 (9)	0.0670 (7)	
H5AA	0.385665	0.295854	0.460393	0.080*	
C6A	0.4299 (2)	0.2718 (3)	0.53333 (9)	0.0593 (6)	
H6AA	0.468889	0.360025	0.540009	0.071*	
C7A	0.6011 (2)	0.3445 (2)	0.67585 (8)	0.0470 (5)	
C8A	0.5983 (2)	0.2358 (2)	0.71298 (8)	0.0454 (5)	
C9A	0.4996 (3)	0.2286 (3)	0.74897 (9)	0.0592 (6)	
H9AA	0.434165	0.293029	0.749979	0.071*	
C10A	0.4954 (3)	0.1287 (3)	0.78346 (9)	0.0607 (6)	
H10B	0.426667	0.126346	0.807108	0.073*	
C11A	0.5920 (2)	0.0304 (2)	0.78387 (8)	0.0458 (5)	
C12A	0.6903 (2)	0.0377 (2)	0.74676 (9)	0.0563 (6)	
H12B	0.755564	−0.026818	0.745152	0.068*	
C13A	0.6926 (2)	0.1388 (3)	0.71237 (9)	0.0570 (6)	
H13B	0.759922	0.141251	0.688154	0.068*	
C14A	0.6685 (2)	0.4666 (2)	0.68521 (9)	0.0545 (6)	
C15A	0.4881 (4)	−0.0761 (3)	0.85675 (12)	0.0900 (10)	
H15D	0.496807	0.005235	0.878115	0.135*	
H15E	0.500050	−0.154259	0.876465	0.135*	
H15F	0.398498	−0.084793	0.840832	0.135*	
C16A	0.6869 (3)	−0.1722 (3)	0.81753 (12)	0.0777 (8)	
H16D	0.778592	−0.131172	0.822915	0.117*	
H16E	0.680026	−0.219876	0.785322	0.117*	
H16F	0.666243	−0.235499	0.843298	0.117*	
N1A	0.47860 (19)	0.2058 (2)	0.62087 (7)	0.0521 (5)	
N2A	0.53735 (18)	0.3236 (2)	0.62771 (7)	0.0494 (4)	
N3A	0.5912 (2)	−0.0680 (2)	0.81912 (7)	0.0600 (5)	
Cl1A	0.25859 (11)	0.05792 (10)	0.41636 (3)	0.1059 (3)	
Cl2A	0.67447 (7)	0.59912 (7)	0.64516 (3)	0.07071 (19)	
Cl3A	0.76185 (9)	0.50455 (7)	0.73961 (3)	0.0824 (2)	
C1B	0.8791 (2)	0.7795 (2)	0.30878 (8)	0.0534 (5)	
C2B	0.9091 (3)	0.8881 (3)	0.34212 (10)	0.0732 (8)	
H2BA	0.907166	0.874525	0.376414	0.088*	
C3B	0.9419 (3)	1.0168 (3)	0.32550 (10)	0.0753 (8)	
H3BA	0.961150	1.090166	0.348319	0.090*	
C4B	0.9458 (3)	1.0357 (3)	0.27501 (9)	0.0580 (6)	
C5B	0.9201 (3)	0.9284 (3)	0.24133 (10)	0.0740 (8)	
H5BA	0.925262	0.942155	0.207103	0.089*	
C6B	0.8865 (3)	0.8002 (3)	0.25782 (9)	0.0710 (7)	
H6BA	0.868609	0.727134	0.234787	0.085*	
C7B	0.7467 (2)	0.4383 (2)	0.31889 (9)	0.0555 (6)	
C8B	0.7881 (2)	0.4045 (2)	0.37050 (9)	0.0496 (5)	
C9B	0.9197 (2)	0.3708 (3)	0.38160 (9)	0.0582 (6)	
H9BA	0.983240	0.368642	0.356103	0.070*	
C10B	0.9597 (2)	0.3403 (3)	0.42917 (10)	0.0609 (6)	
H10C	1.048949	0.315983	0.434946	0.073*	
C11B	0.8701 (2)	0.3447 (2)	0.46910 (9)	0.0527 (5)	
C12B	0.7377 (3)	0.3785 (3)	0.45733 (11)	0.0753 (8)	
H12C	0.673596	0.382026	0.482612	0.090*	
C13B	0.6991 (3)	0.4069 (3)	0.40940 (11)	0.0752 (8)	
H13C	0.609160	0.428491	0.403106	0.090*	
C14B	0.6730 (3)	0.3507 (3)	0.28757 (10)	0.0675 (7)	
C15B	1.0461 (3)	0.2825 (4)	0.52857 (13)	0.1025 (12)	
H15G	1.111103	0.339811	0.510989	0.154*	
H15H	1.054616	0.188677	0.518344	0.154*	
H15I	1.064191	0.294754	0.564051	0.154*	
C16B	0.8219 (3)	0.3376 (3)	0.55890 (11)	0.0845 (9)	
H16G	0.785760	0.425916	0.557867	0.127*	
H16H	0.873595	0.331524	0.589666	0.127*	
H16I	0.747446	0.268088	0.557111	0.127*	
N1B	0.8435 (2)	0.6513 (2)	0.32988 (7)	0.0580 (5)	
N2B	0.7831 (2)	0.5669 (2)	0.29885 (7)	0.0582 (5)	
N3B	0.9100 (2)	0.3187 (3)	0.51715 (8)	0.0759 (7)	
Cl1B	0.98597 (9)	1.19767 (8)	0.25356 (3)	0.0849 (2)	
Cl2B	0.62580 (10)	0.38856 (10)	0.22766 (3)	0.1031 (3)	
Cl3B	0.61794 (9)	0.18941 (8)	0.30326 (3)	0.0901 (2)	

### Atomic displacement parameters (Å^2^)

**Table:**

	*U*^11^	*U*^22^	*U*^33^	*U*^12^	*U*^13^	*U*^23^
C1	0.0467 (12)	0.0453 (12)	0.0428 (11)	−0.0008 (9)	−0.0046 (9)	0.0019 (9)
C2	0.0661 (15)	0.0469 (13)	0.0481 (13)	−0.0105 (11)	−0.0127 (11)	0.0010 (10)
C3	0.0721 (16)	0.0548 (14)	0.0428 (12)	−0.0086 (11)	−0.0103 (11)	0.0064 (10)
C4	0.0547 (13)	0.0455 (12)	0.0560 (14)	−0.0045 (10)	−0.0061 (11)	0.0098 (10)
C5	0.0580 (14)	0.0462 (13)	0.0553 (14)	−0.0055 (10)	−0.0114 (11)	−0.0032 (10)
C6	0.0567 (14)	0.0524 (13)	0.0424 (12)	−0.0024 (10)	−0.0075 (10)	−0.0024 (10)
C7	0.0461 (12)	0.0515 (13)	0.0446 (12)	−0.0005 (10)	0.0033 (9)	0.0038 (10)
C8	0.0452 (12)	0.0471 (12)	0.0410 (11)	−0.0005 (9)	−0.0003 (9)	0.0058 (9)
C9	0.0513 (13)	0.0570 (14)	0.0520 (13)	0.0034 (11)	0.0091 (10)	−0.0011 (11)
C10	0.0423 (12)	0.0613 (14)	0.0627 (14)	−0.0025 (10)	0.0053 (11)	0.0012 (12)
C11	0.0527 (13)	0.0430 (12)	0.0535 (13)	0.0019 (10)	−0.0043 (10)	0.0066 (10)
C12	0.0535 (14)	0.0487 (13)	0.0541 (13)	0.0097 (10)	0.0019 (10)	−0.0009 (10)
C13	0.0428 (12)	0.0542 (13)	0.0556 (13)	0.0053 (10)	0.0030 (10)	0.0055 (11)
C14	0.0527 (13)	0.0564 (13)	0.0422 (12)	−0.0051 (10)	0.0016 (10)	0.0009 (10)
C15	0.0653 (18)	0.080 (2)	0.116 (3)	−0.0120 (15)	−0.0196 (17)	−0.0149 (18)
C16	0.097 (2)	0.0678 (18)	0.0799 (19)	−0.0049 (16)	−0.0045 (16)	−0.0200 (15)
N1	0.0551 (11)	0.0494 (11)	0.0456 (11)	−0.0032 (8)	−0.0037 (8)	0.0037 (8)
N2	0.0545 (11)	0.0534 (11)	0.0434 (10)	−0.0046 (9)	−0.0012 (8)	0.0041 (8)
N3	0.0592 (13)	0.0619 (13)	0.0931 (17)	−0.0044 (10)	−0.0078 (12)	−0.0172 (12)
Cl1	0.1108 (6)	0.0707 (4)	0.0783 (5)	−0.0343 (4)	−0.0217 (4)	0.0297 (4)
Cl2	0.0924 (5)	0.0755 (4)	0.0505 (3)	−0.0260 (4)	−0.0073 (3)	−0.0010 (3)
Cl3	0.0757 (4)	0.0640 (4)	0.0483 (3)	−0.0032 (3)	0.0018 (3)	0.0122 (3)
C1A	0.0444 (12)	0.0593 (14)	0.0454 (12)	0.0022 (10)	−0.0015 (9)	0.0029 (10)
C2A	0.0755 (17)	0.0601 (15)	0.0561 (15)	0.0021 (13)	−0.0064 (12)	0.0062 (12)
C3A	0.0817 (19)	0.0587 (16)	0.0729 (18)	0.0033 (13)	−0.0159 (14)	−0.0100 (14)
C4A	0.0644 (16)	0.0774 (18)	0.0519 (14)	0.0146 (13)	−0.0122 (12)	−0.0087 (13)
C5A	0.0687 (17)	0.0817 (19)	0.0502 (14)	0.0011 (14)	−0.0101 (12)	0.0115 (13)
C6A	0.0601 (15)	0.0648 (15)	0.0511 (14)	−0.0068 (12)	−0.0070 (11)	0.0054 (11)
C7A	0.0484 (12)	0.0537 (13)	0.0393 (11)	0.0049 (10)	−0.0018 (9)	0.0048 (9)
C8A	0.0477 (12)	0.0499 (12)	0.0381 (11)	0.0014 (10)	−0.0023 (9)	0.0003 (9)
C9A	0.0627 (15)	0.0602 (15)	0.0577 (14)	0.0197 (12)	0.0111 (12)	0.0090 (12)
C10A	0.0650 (15)	0.0648 (15)	0.0552 (14)	0.0146 (12)	0.0180 (12)	0.0106 (12)
C11A	0.0514 (13)	0.0446 (12)	0.0403 (11)	−0.0015 (9)	−0.0049 (9)	−0.0003 (9)
C12A	0.0567 (14)	0.0564 (14)	0.0581 (14)	0.0158 (11)	0.0053 (11)	0.0069 (11)
C13A	0.0566 (14)	0.0644 (15)	0.0521 (13)	0.0110 (12)	0.0118 (11)	0.0103 (11)
C14A	0.0598 (14)	0.0541 (14)	0.0492 (13)	0.0008 (11)	−0.0056 (11)	0.0072 (10)
C15A	0.117 (3)	0.080 (2)	0.0776 (19)	0.0204 (18)	0.0359 (19)	0.0309 (16)
C16A	0.095 (2)	0.0595 (16)	0.0822 (19)	0.0187 (15)	0.0033 (16)	0.0202 (14)
N1A	0.0521 (11)	0.0590 (12)	0.0448 (10)	−0.0002 (9)	−0.0017 (8)	0.0048 (9)
N2A	0.0486 (10)	0.0559 (11)	0.0437 (10)	0.0022 (9)	−0.0021 (8)	0.0050 (8)
N3A	0.0739 (14)	0.0538 (12)	0.0542 (11)	0.0107 (10)	0.0075 (10)	0.0126 (9)
Cl1A	0.1388 (8)	0.1108 (7)	0.0653 (5)	0.0166 (6)	−0.0417 (5)	−0.0205 (4)
Cl2A	0.0811 (4)	0.0591 (4)	0.0719 (4)	−0.0015 (3)	−0.0089 (3)	0.0195 (3)
Cl3A	0.1102 (6)	0.0644 (4)	0.0681 (4)	−0.0145 (4)	−0.0345 (4)	0.0034 (3)
C1B	0.0573 (14)	0.0551 (14)	0.0474 (13)	0.0008 (11)	−0.0043 (10)	0.0054 (10)
C2B	0.117 (2)	0.0571 (16)	0.0454 (13)	0.0029 (15)	−0.0056 (14)	0.0047 (12)
C3B	0.118 (2)	0.0528 (15)	0.0550 (15)	0.0041 (15)	0.0002 (15)	−0.0007 (12)
C4B	0.0583 (14)	0.0583 (14)	0.0585 (15)	0.0047 (11)	0.0070 (11)	0.0125 (12)
C5B	0.093 (2)	0.0810 (19)	0.0453 (14)	−0.0183 (16)	0.0008 (13)	0.0091 (13)
C6B	0.091 (2)	0.0711 (17)	0.0465 (14)	−0.0179 (14)	−0.0012 (13)	−0.0052 (12)
C7B	0.0515 (13)	0.0519 (13)	0.0622 (15)	0.0031 (10)	−0.0065 (11)	−0.0017 (11)
C8B	0.0470 (13)	0.0444 (12)	0.0568 (13)	0.0016 (9)	−0.0025 (10)	0.0011 (10)
C9B	0.0513 (14)	0.0680 (16)	0.0565 (14)	0.0114 (11)	0.0065 (11)	0.0015 (12)
C10B	0.0452 (13)	0.0731 (17)	0.0660 (16)	0.0132 (11)	0.0021 (11)	0.0061 (13)
C11B	0.0482 (13)	0.0515 (13)	0.0590 (14)	0.0045 (10)	0.0043 (11)	0.0063 (11)
C12B	0.0498 (15)	0.111 (2)	0.0682 (17)	0.0185 (15)	0.0148 (13)	0.0153 (16)
C13B	0.0464 (14)	0.105 (2)	0.0774 (19)	0.0201 (14)	0.0014 (13)	0.0136 (16)
C14B	0.0640 (16)	0.0637 (16)	0.0731 (17)	0.0020 (12)	−0.0146 (13)	−0.0041 (13)
C15B	0.083 (2)	0.152 (3)	0.079 (2)	0.037 (2)	−0.0014 (17)	0.037 (2)
C16B	0.095 (2)	0.099 (2)	0.0606 (17)	0.0094 (18)	0.0140 (16)	−0.0004 (16)
N1B	0.0681 (13)	0.0522 (12)	0.0527 (11)	0.0000 (10)	−0.0072 (10)	0.0010 (9)
N2B	0.0605 (12)	0.0570 (12)	0.0560 (12)	0.0011 (9)	−0.0080 (9)	−0.0002 (10)
N3B	0.0611 (13)	0.1092 (19)	0.0611 (13)	0.0214 (13)	0.0082 (11)	0.0188 (13)
Cl1B	0.1076 (6)	0.0658 (4)	0.0832 (5)	0.0017 (4)	0.0239 (4)	0.0219 (4)
Cl2B	0.1230 (7)	0.0965 (6)	0.0848 (5)	−0.0064 (5)	−0.0471 (5)	−0.0079 (4)
Cl3B	0.0889 (5)	0.0609 (4)	0.1158 (6)	−0.0135 (4)	−0.0188 (5)	−0.0088 (4)

### Geometric parameters (Å, º)

**Table:**

C1—C2	1.386 (3)	C10A—C11A	1.395 (3)
C1—C6	1.388 (3)	C10A—H10B	0.9300
C1—N1	1.421 (3)	C11A—N3A	1.376 (3)
C2—C3	1.380 (3)	C11A—C12A	1.393 (3)
C2—H2A	0.9300	C12A—C13A	1.378 (3)
C3—C4	1.373 (3)	C12A—H12B	0.9300
C3—H3A	0.9300	C13A—H13B	0.9300
C4—C5	1.377 (3)	C14A—Cl3A	1.710 (2)
C4—Cl1	1.734 (2)	C14A—Cl2A	1.715 (2)
C5—C6	1.378 (3)	C15A—N3A	1.436 (3)
C5—H5A	0.9300	C15A—H15D	0.9600
C6—H6A	0.9300	C15A—H15E	0.9600
C7—C14	1.332 (3)	C15A—H15F	0.9600
C7—N2	1.418 (3)	C16A—N3A	1.433 (3)
C7—C8	1.481 (3)	C16A—H16D	0.9600
C8—C9	1.383 (3)	C16A—H16E	0.9600
C8—C13	1.385 (3)	C16A—H16F	0.9600
C9—C10	1.377 (3)	N1A—N2A	1.259 (3)
C9—H9A	0.9300	C1B—C2B	1.372 (3)
C10—C11	1.393 (3)	C1B—C6B	1.385 (3)
C10—H10A	0.9300	C1B—N1B	1.423 (3)
C11—N3	1.370 (3)	C2B—C3B	1.375 (4)
C11—C12	1.403 (3)	C2B—H2BA	0.9300
C12—C13	1.368 (3)	C3B—C4B	1.368 (4)
C12—H12A	0.9300	C3B—H3BA	0.9300
C13—H13A	0.9300	C4B—C5B	1.362 (4)
C14—Cl3	1.713 (2)	C4B—Cl1B	1.734 (2)
C14—Cl2	1.714 (2)	C5B—C6B	1.370 (4)
C15—N3	1.445 (4)	C5B—H5BA	0.9300
C15—H15A	0.9600	C6B—H6BA	0.9300
C15—H15B	0.9600	C7B—C14B	1.335 (3)
C15—H15C	0.9600	C7B—N2B	1.417 (3)
C16—N3	1.436 (3)	C7B—C8B	1.483 (3)
C16—H16A	0.9600	C8B—C13B	1.367 (3)
C16—H16B	0.9600	C8B—C9B	1.378 (3)
C16—H16C	0.9600	C9B—C10B	1.370 (3)
N1—N2	1.257 (2)	C9B—H9BA	0.9300
C1A—C2A	1.378 (3)	C10B—C11B	1.393 (3)
C1A—C6A	1.386 (3)	C10B—H10C	0.9300
C1A—N1A	1.425 (3)	C11B—N3B	1.370 (3)
C2A—C3A	1.387 (4)	C11B—C12B	1.389 (3)
C2A—H2AA	0.9300	C12B—C13B	1.370 (4)
C3A—C4A	1.370 (4)	C12B—H12C	0.9300
C3A—H3AA	0.9300	C13B—H13C	0.9300
C4A—C5A	1.371 (4)	C14B—Cl3B	1.707 (3)
C4A—Cl1A	1.733 (3)	C14B—Cl2B	1.716 (3)
C5A—C6A	1.374 (3)	C15B—N3B	1.428 (4)
C5A—H5AA	0.9300	C15B—H15G	0.9600
C6A—H6AA	0.9300	C15B—H15H	0.9600
C7A—C14A	1.338 (3)	C15B—H15I	0.9600
C7A—N2A	1.415 (3)	C16B—N3B	1.435 (3)
C7A—C8A	1.484 (3)	C16B—H16G	0.9600
C8A—C13A	1.370 (3)	C16B—H16H	0.9600
C8A—C9A	1.374 (3)	C16B—H16I	0.9600
C9A—C10A	1.371 (3)	N1B—N2B	1.255 (3)
C9A—H9AA	0.9300		
			
C2—C1—C6	119.6 (2)	N3A—C11A—C12A	121.6 (2)
C2—C1—N1	115.70 (19)	N3A—C11A—C10A	122.0 (2)
C6—C1—N1	124.67 (19)	C12A—C11A—C10A	116.4 (2)
C3—C2—C1	120.6 (2)	C13A—C12A—C11A	121.3 (2)
C3—C2—H2A	119.7	C13A—C12A—H12B	119.4
C1—C2—H2A	119.7	C11A—C12A—H12B	119.4
C4—C3—C2	118.8 (2)	C8A—C13A—C12A	121.6 (2)
C4—C3—H3A	120.6	C8A—C13A—H13B	119.2
C2—C3—H3A	120.6	C12A—C13A—H13B	119.2
C3—C4—C5	121.6 (2)	C7A—C14A—Cl3A	122.02 (18)
C3—C4—Cl1	119.95 (18)	C7A—C14A—Cl2A	125.13 (18)
C5—C4—Cl1	118.43 (18)	Cl3A—C14A—Cl2A	112.84 (14)
C4—C5—C6	119.4 (2)	N3A—C15A—H15D	109.5
C4—C5—H5A	120.3	N3A—C15A—H15E	109.5
C6—C5—H5A	120.3	H15D—C15A—H15E	109.5
C5—C6—C1	120.0 (2)	N3A—C15A—H15F	109.5
C5—C6—H6A	120.0	H15D—C15A—H15F	109.5
C1—C6—H6A	120.0	H15E—C15A—H15F	109.5
C14—C7—N2	115.2 (2)	N3A—C16A—H16D	109.5
C14—C7—C8	123.2 (2)	N3A—C16A—H16E	109.5
N2—C7—C8	121.58 (18)	H16D—C16A—H16E	109.5
C9—C8—C13	117.1 (2)	N3A—C16A—H16F	109.5
C9—C8—C7	121.4 (2)	H16D—C16A—H16F	109.5
C13—C8—C7	121.44 (19)	H16E—C16A—H16F	109.5
C10—C9—C8	121.7 (2)	N2A—N1A—C1A	113.44 (18)
C10—C9—H9A	119.2	N1A—N2A—C7A	112.75 (18)
C8—C9—H9A	119.2	C11A—N3A—C16A	121.3 (2)
C9—C10—C11	121.4 (2)	C11A—N3A—C15A	121.2 (2)
C9—C10—H10A	119.3	C16A—N3A—C15A	117.4 (2)
C11—C10—H10A	119.3	C2B—C1B—C6B	119.0 (2)
N3—C11—C10	122.3 (2)	C2B—C1B—N1B	116.4 (2)
N3—C11—C12	121.2 (2)	C6B—C1B—N1B	124.6 (2)
C10—C11—C12	116.5 (2)	C1B—C2B—C3B	120.9 (2)
C13—C12—C11	121.3 (2)	C1B—C2B—H2BA	119.6
C13—C12—H12A	119.3	C3B—C2B—H2BA	119.6
C11—C12—H12A	119.3	C4B—C3B—C2B	119.2 (3)
C12—C13—C8	121.9 (2)	C4B—C3B—H3BA	120.4
C12—C13—H13A	119.0	C2B—C3B—H3BA	120.4
C8—C13—H13A	119.0	C5B—C4B—C3B	120.8 (2)
C7—C14—Cl3	122.13 (18)	C5B—C4B—Cl1B	119.55 (19)
C7—C14—Cl2	124.47 (18)	C3B—C4B—Cl1B	119.7 (2)
Cl3—C14—Cl2	113.40 (13)	C4B—C5B—C6B	120.1 (2)
N3—C15—H15A	109.5	C4B—C5B—H5BA	120.0
N3—C15—H15B	109.5	C6B—C5B—H5BA	120.0
H15A—C15—H15B	109.5	C5B—C6B—C1B	120.0 (2)
N3—C15—H15C	109.5	C5B—C6B—H6BA	120.0
H15A—C15—H15C	109.5	C1B—C6B—H6BA	120.0
H15B—C15—H15C	109.5	C14B—C7B—N2B	114.6 (2)
N3—C16—H16A	109.5	C14B—C7B—C8B	123.1 (2)
N3—C16—H16B	109.5	N2B—C7B—C8B	122.3 (2)
H16A—C16—H16B	109.5	C13B—C8B—C9B	116.7 (2)
N3—C16—H16C	109.5	C13B—C8B—C7B	122.1 (2)
H16A—C16—H16C	109.5	C9B—C8B—C7B	121.2 (2)
H16B—C16—H16C	109.5	C10B—C9B—C8B	121.8 (2)
N2—N1—C1	113.53 (18)	C10B—C9B—H9BA	119.1
N1—N2—C7	113.61 (18)	C8B—C9B—H9BA	119.1
C11—N3—C16	121.5 (2)	C9B—C10B—C11B	121.7 (2)
C11—N3—C15	120.0 (2)	C9B—C10B—H10C	119.1
C16—N3—C15	117.8 (2)	C11B—C10B—H10C	119.1
C2A—C1A—C6A	119.2 (2)	N3B—C11B—C12B	121.9 (2)
C2A—C1A—N1A	115.7 (2)	N3B—C11B—C10B	122.3 (2)
C6A—C1A—N1A	125.0 (2)	C12B—C11B—C10B	115.8 (2)
C1A—C2A—C3A	120.4 (2)	C13B—C12B—C11B	121.6 (2)
C1A—C2A—H2AA	119.8	C13B—C12B—H12C	119.2
C3A—C2A—H2AA	119.8	C11B—C12B—H12C	119.2
C4A—C3A—C2A	119.2 (3)	C8B—C13B—C12B	122.3 (2)
C4A—C3A—H3AA	120.4	C8B—C13B—H13C	118.8
C2A—C3A—H3AA	120.4	C12B—C13B—H13C	118.8
C3A—C4A—C5A	121.2 (2)	C7B—C14B—Cl3B	123.1 (2)
C3A—C4A—Cl1A	119.9 (2)	C7B—C14B—Cl2B	123.4 (2)
C5A—C4A—Cl1A	118.9 (2)	Cl3B—C14B—Cl2B	113.56 (15)
C4A—C5A—C6A	119.4 (2)	N3B—C15B—H15G	109.5
C4A—C5A—H5AA	120.3	N3B—C15B—H15H	109.5
C6A—C5A—H5AA	120.3	H15G—C15B—H15H	109.5
C5A—C6A—C1A	120.5 (2)	N3B—C15B—H15I	109.5
C5A—C6A—H6AA	119.7	H15G—C15B—H15I	109.5
C1A—C6A—H6AA	119.7	H15H—C15B—H15I	109.5
C14A—C7A—N2A	115.90 (19)	N3B—C16B—H16G	109.5
C14A—C7A—C8A	121.9 (2)	N3B—C16B—H16H	109.5
N2A—C7A—C8A	122.12 (19)	H16G—C16B—H16H	109.5
C13A—C8A—C9A	117.7 (2)	N3B—C16B—H16I	109.5
C13A—C8A—C7A	121.45 (19)	H16G—C16B—H16I	109.5
C9A—C8A—C7A	120.8 (2)	H16H—C16B—H16I	109.5
C10A—C9A—C8A	121.5 (2)	N2B—N1B—C1B	112.63 (19)
C10A—C9A—H9AA	119.2	N1B—N2B—C7B	113.78 (19)
C8A—C9A—H9AA	119.2	C11B—N3B—C15B	121.2 (2)
C9A—C10A—C11A	121.5 (2)	C11B—N3B—C16B	122.0 (2)
C9A—C10A—H10B	119.3	C15B—N3B—C16B	116.6 (2)
C11A—C10A—H10B	119.3		
			
C6—C1—C2—C3	1.1 (4)	N3A—C11A—C12A—C13A	−178.3 (2)
N1—C1—C2—C3	−179.3 (2)	C10A—C11A—C12A—C13A	1.3 (4)
C1—C2—C3—C4	−0.4 (4)	C9A—C8A—C13A—C12A	−0.5 (4)
C2—C3—C4—C5	−0.6 (4)	C7A—C8A—C13A—C12A	−180.0 (2)
C2—C3—C4—Cl1	179.58 (19)	C11A—C12A—C13A—C8A	−0.4 (4)
C3—C4—C5—C6	0.9 (4)	N2A—C7A—C14A—Cl3A	−175.07 (17)
Cl1—C4—C5—C6	−179.27 (18)	C8A—C7A—C14A—Cl3A	2.5 (3)
C4—C5—C6—C1	−0.2 (4)	N2A—C7A—C14A—Cl2A	3.8 (3)
C2—C1—C6—C5	−0.8 (3)	C8A—C7A—C14A—Cl2A	−178.64 (17)
N1—C1—C6—C5	179.7 (2)	C2A—C1A—N1A—N2A	177.3 (2)
C14—C7—C8—C9	74.0 (3)	C6A—C1A—N1A—N2A	−0.3 (3)
N2—C7—C8—C9	−105.6 (2)	C1A—N1A—N2A—C7A	−175.50 (18)
C14—C7—C8—C13	−108.2 (3)	C14A—C7A—N2A—N1A	178.4 (2)
N2—C7—C8—C13	72.2 (3)	C8A—C7A—N2A—N1A	0.8 (3)
C13—C8—C9—C10	−1.6 (3)	C12A—C11A—N3A—C16A	−3.5 (4)
C7—C8—C9—C10	176.3 (2)	C10A—C11A—N3A—C16A	176.9 (3)
C8—C9—C10—C11	0.5 (4)	C12A—C11A—N3A—C15A	−179.0 (3)
C9—C10—C11—N3	−175.9 (2)	C10A—C11A—N3A—C15A	1.4 (4)
C9—C10—C11—C12	1.3 (3)	C6B—C1B—C2B—C3B	2.0 (4)
N3—C11—C12—C13	175.3 (2)	N1B—C1B—C2B—C3B	−178.7 (3)
C10—C11—C12—C13	−2.0 (3)	C1B—C2B—C3B—C4B	−0.7 (5)
C11—C12—C13—C8	1.0 (3)	C2B—C3B—C4B—C5B	−1.1 (4)
C9—C8—C13—C12	0.9 (3)	C2B—C3B—C4B—Cl1B	179.6 (2)
C7—C8—C13—C12	−177.0 (2)	C3B—C4B—C5B—C6B	1.6 (4)
N2—C7—C14—Cl3	−176.21 (16)	Cl1B—C4B—C5B—C6B	−179.1 (2)
C8—C7—C14—Cl3	4.1 (3)	C4B—C5B—C6B—C1B	−0.2 (4)
N2—C7—C14—Cl2	3.1 (3)	C2B—C1B—C6B—C5B	−1.6 (4)
C8—C7—C14—Cl2	−176.55 (17)	N1B—C1B—C6B—C5B	179.2 (3)
C2—C1—N1—N2	173.7 (2)	C14B—C7B—C8B—C13B	−78.7 (3)
C6—C1—N1—N2	−6.7 (3)	N2B—C7B—C8B—C13B	101.5 (3)
C1—N1—N2—C7	−179.12 (18)	C14B—C7B—C8B—C9B	102.2 (3)
C14—C7—N2—N1	−178.7 (2)	N2B—C7B—C8B—C9B	−77.6 (3)
C8—C7—N2—N1	1.0 (3)	C13B—C8B—C9B—C10B	0.4 (4)
C10—C11—N3—C16	−172.1 (2)	C7B—C8B—C9B—C10B	179.5 (2)
C12—C11—N3—C16	10.8 (4)	C8B—C9B—C10B—C11B	−1.4 (4)
C10—C11—N3—C15	−1.9 (4)	C9B—C10B—C11B—N3B	−177.9 (3)
C12—C11—N3—C15	−179.0 (2)	C9B—C10B—C11B—C12B	1.5 (4)
C6A—C1A—C2A—C3A	0.4 (4)	N3B—C11B—C12B—C13B	178.8 (3)
N1A—C1A—C2A—C3A	−177.3 (2)	C10B—C11B—C12B—C13B	−0.6 (4)
C1A—C2A—C3A—C4A	0.9 (4)	C9B—C8B—C13B—C12B	0.6 (4)
C2A—C3A—C4A—C5A	−1.4 (4)	C7B—C8B—C13B—C12B	−178.6 (3)
C2A—C3A—C4A—Cl1A	179.1 (2)	C11B—C12B—C13B—C8B	−0.4 (5)
C3A—C4A—C5A—C6A	0.5 (4)	N2B—C7B—C14B—Cl3B	179.71 (18)
Cl1A—C4A—C5A—C6A	−179.9 (2)	C8B—C7B—C14B—Cl3B	−0.1 (4)
C4A—C5A—C6A—C1A	0.8 (4)	N2B—C7B—C14B—Cl2B	0.3 (3)
C2A—C1A—C6A—C5A	−1.2 (4)	C8B—C7B—C14B—Cl2B	−179.54 (19)
N1A—C1A—C6A—C5A	176.3 (2)	C2B—C1B—N1B—N2B	162.2 (2)
C14A—C7A—C8A—C13A	−92.7 (3)	C6B—C1B—N1B—N2B	−18.5 (3)
N2A—C7A—C8A—C13A	84.7 (3)	C1B—N1B—N2B—C7B	179.93 (19)
C14A—C7A—C8A—C9A	87.8 (3)	C14B—C7B—N2B—N1B	175.0 (2)
N2A—C7A—C8A—C9A	−94.7 (3)	C8B—C7B—N2B—N1B	−5.2 (3)
C13A—C8A—C9A—C10A	0.4 (4)	C12B—C11B—N3B—C15B	179.3 (3)
C7A—C8A—C9A—C10A	179.9 (2)	C10B—C11B—N3B—C15B	−1.3 (4)
C8A—C9A—C10A—C11A	0.6 (4)	C12B—C11B—N3B—C16B	−6.2 (4)
C9A—C10A—C11A—N3A	178.2 (2)	C10B—C11B—N3B—C16B	173.2 (3)
C9A—C10A—C11A—C12A	−1.4 (4)		

### Hydrogen-bond geometry (Å, º)

*Cg*1 and *Cg*4 are the centroids of the C1–C6 and C8*A*–C13*A* rings, respectively.

**Table:**

*D*—H···*A*	*D*—H	H···*A*	*D*···*A*	*D*—H···*A*
C2—H2*A*···N3*A*	0.93	2.68	3.597 (3)	167
C5*B*—H5*BA*···Cl3^i^	0.93	2.95	3.703 (3)	139
C14—Cl3···*Cg*1^ii^	1.71 (1)	3.55 (1)	4.083 (2)	96 (1)
C14*B*—Cl3*B*···*Cg*4^iii^	1.71 (1)	3.85 (1)	5.300 (3)	142 (1)

Symmetry codes: (i) *x*, *y*+1, *z*−1; (ii) −*x*+2, −*y*, −*z*+2; (iii) −*x*+1, −*y*, −*z*+1.

## References

[bb1] Afkhami, F. A., Mahmoudi, G., Gurbanov, A. V., Zubkov, F. I., Qu, F., Gupta, A. & Safin, D. A. (2017). *Dalton Trans.* **46**, 14888–14896. 10.1039/c7dt02952g 29043338

[bb2] Akkurt, M., Shikhaliyev, N. Q., Suleymanova, G. T., Babayeva, G. V., Mammadova, G. Z., Niyazova, A. A., Shikhaliyeva, I. M. & Toze, F. A. A. (2019). *Acta Cryst.* E**75**, 1199–1204. 10.1107/S2056989019010004 PMC6690453 31417792

[bb3] Asadov, Z. H., Rahimov, R. A., Ahmadova, G. A., Mammadova, K. A. & Gurbanov, A. V. (2016). *J. Surfactants Deterg.* **19**, 145–153.

[bb4] Atioğlu, Z., Akkurt, M., Shikhaliyev, N. Q., Suleymanova, G. T., Bagirova, K. N. & Toze, F. A. A. (2019). *Acta Cryst.* E**75**, 237–241. 10.1107/S2056989019000707 PMC6362658 30800458

[bb5] Bruker (2007). *APEX2* and *SAINT*. Bruker AXS Inc., Madison, Wisconsin, USA.

[bb6] Dolomanov, O. V., Bourhis, L. J., Gildea, R. J., Howard, J. A. K. & Puschmann, H. (2009). *J. Appl. Cryst.* **42**, 339–341.

[bb7] Farrugia, L. J. (2012). *J. Appl. Cryst.* **45**, 849–854.

[bb8] Groom, C. R., Bruno, I. J., Lightfoot, M. P. & Ward, S. C. (2016). *Acta Cryst.* B**72**, 171–179. 10.1107/S2052520616003954 PMC4822653 27048719

[bb9] Gurbanov, A. V., Maharramov, A. M., Zubkov, F. I., Saifutdinov, A. M. & Guseinov, F. I. (2018). *Aust. J. Chem.* **71**, 190–194.

[bb10] Krause, L., Herbst-Irmer, R., Sheldrick, G. M. & Stalke, D. (2015). *J. Appl. Cryst.* **48**, 3–10. 10.1107/S1600576714022985 PMC4453166 26089746

[bb11] Maharramov, A. M., Shikhaliyev, N. Q., Suleymanova, G. T., Gurbanov, A. V., Babayeva, G. V., Mammadova, G. Z., Zubkov, F. I., Nenajdenko, V. G., Mahmudov, K. T. & Pombeiro, A. J. L. (2018). *Dyes Pigments*, **159**, 135–141.

[bb12] Mahmudov, K. T., Gurbanov, A. V., Guseinov, F. I. & Guedes da Silva, M. F. C. (2019). *Coord. Chem. Rev.* **387**, 32–46.

[bb13] Mahmudov, K. T. & Pombeiro, A. J. L. (2016). *Chem. Eur. J.* **22**, 16356–16398. 10.1002/chem.201601766 27492126

[bb14] McKinnon, J. J., Jayatilaka, D. & Spackman, M. A. (2007). *Chem. Commun.* pp. 3814–3816. 10.1039/b704980c 18217656

[bb15] Sheldrick, G. M. (2015*a*). *Acta Cryst.* A**71**, 3–8.

[bb16] Sheldrick, G. M. (2015*b*). *Acta Cryst.* C**71**, 3–8.

[bb17] Shikhaliyev, N. Q., Ahmadova, N. E., Gurbanov, A. V., Maharramov, A. M., Mammadova, G. Z., Nenajdenko, V. G., Zubkov, F. I., Mahmudov, K. T. & Pombeiro, A. J. L. (2018). *Dyes Pigments*, **150**, 377–381.

[bb18] Shikhaliyev, N. Q., Çelikesir, S. T., Akkurt, M., Bagirova, K. N., Suleymanova, G. T. & Toze, F. A. A. (2019). *Acta Cryst.* E**75**, 465–469. 10.1107/S2056989019003657 PMC6509676 31161058

[bb19] Spackman, M. A. & Jayatilaka, D. (2009). *CrystEngComm*, **11**, 19–32.

[bb20] Spek, A. L. (2020). *Acta Cryst.* E**76**, 1–11. 10.1107/S2056989019016244 PMC6944088 31921444

[bb21] Turner, M. J., McKinnon, J. J., Wolff, S. K., Grimwood, D. J., Spackman, P. R., Jayatilaka, D. & Spackman, M. A. (2017). CrystalExplorer17. The University of Western Australia.

[bb22] Westrip, S. P. (2010). *J. Appl. Cryst.* **43**, 920–925.

